# HER2 + breast cancers evade anti-HER2 therapy via a switch in driver pathway

**DOI:** 10.1038/s41467-021-27093-y

**Published:** 2021-11-18

**Authors:** Alison E. Smith, Emanuela Ferraro, Anton Safonov, Cristina Bernado Morales, Enrique J. Arenas Lahuerta, Qing Li, Amanda Kulick, Dara Ross, David B. Solit, Elisa de Stanchina, Jorge Reis-Filho, Neal Rosen, Joaquín Arribas, Pedram Razavi, Sarat Chandarlapaty

**Affiliations:** 1 Human Oncology and Pathogenesis Program (HOPP), Memorial Sloan Kettering Cancer Center, New York, NY 10065 USA; 2 Weill Cornell Medicine, New York, NY 10065 USA; 3 Breast Medicine Service, Department of Medicine, Memorial Sloan Kettering Cancer Center, New York, NY 10065 USA; 4 Preclinical Research Program, Vall d’Hebron Institute of Oncology, Barcelona, Spain; 5 Antitumor Assessment Core, Memorial Sloan Kettering Cancer Center, New York, NY 10065 USA; 6 Department of Pathology, Memorial Sloan Kettering Cancer Center, New York, NY 10065 USA; 7 Molecular Pharmacology and Chemistry Program and Center for Cell Engineering, Memorial Sloan Kettering Cancer Center, New York, NY 10065 USA

**Keywords:** Breast cancer, Growth factor signalling, Cancer therapeutic resistance

## Abstract

Inhibition of HER2 in HER2-amplified breast cancer has been remarkably successful clinically, as demonstrated by the efficacy of HER-kinase inhibitors and HER2-antibody treatments. Whilst resistance to HER2 inhibition is common in the metastatic setting, the specific programs downstream of HER2 driving resistance are not established. Through genomic profiling of 733 HER2-amplified breast cancers, we identify enrichment of somatic alterations that promote MEK/ERK signaling in metastatic tumors with shortened progression-free survival on anti-HER2 therapy. These mutations, including *NF1* loss and *ERBB2* activating mutations, are sufficient to mediate resistance to FDA-approved HER2 kinase inhibitors including tucatinib and neratinib. Moreover, resistant tumors lose AKT dependence while undergoing a dramatic sensitization to MEK/ERK inhibition. Mechanistically, this driver pathway switch is a result of MEK-dependent activation of CDK2 kinase. These results establish genetic activation of MAPK as a recurrent mechanism of anti-HER2 therapy resistance that may be effectively combated with MEK/ERK inhibitors.

## Abstract (web summary)

Resistance to HER2 inhibition in HER2- amplified breast cancer is common in the metastatic setting and the underlying molecular mechanisms remain unknown. Here, the authors, perform genomic profiling of 733 HER2-amplified breast cancers and propose genetic activation of MAPK as a resistance mechanism.

## Introduction

The discovery and pharmacologic inhibition of the HER2/neu oncogene in breast cancer represents a hallmark success in targeted therapy in oncology. The efficacy of HER2-targeted therapies specifically hinges upon their ability to inhibit PI3K signaling^1^, despite activation of multiple other pathways by the receptor, including the ERK pathway. Indeed, PI3K/AKT inhibitors, but not MEK/ERK inhibitors, have major antitumor effects in models of HER2-amplified breast cancer^2–5^. This marked pathway dependence has been attributed to the entrainment of multiple cell cycle regulators including cyclin D1 and p27 by AKT in this context^6–12^. More recently, combinations of anti-HER2 therapies together with additional drugs to downregulate the PI3K/AKT pathway (pertuzumab, alpelisib, everolimus) have been advanced in the clinic on the basis of this work.

Despite the clinical success of HER2/HER3/PI3K-targeted therapies in breast cancer treatment, de novo and acquired resistance nonetheless occurs, particularly in the metastatic setting^13,14^. Both preclinical and early clinical data have suggested that activating mutations in the PI3K pathway (*PIK3CA* mutation, *PTEN* loss, or *ERBB3* mutation) reduce the efficacy of anti-HER2 treatments^15–20^. Moreover, genomic alterations in the cell regulators p27^21^, cyclin E^22^, and cyclin D/CDK4^23^ have also been implicated in resistance to HER2-targeted therapies. However, these alterations have proven neither binary markers of sensitivity nor sufficiently comprehensive to elucidate the basis for resistance in the majority of patients.

Here, we define the genomic landscape of HER2 + breast cancer including both primary, treatment-naïve tumors as well as anti-HER2 treatment-refractory metastatic tumors. Intriguingly, we find an enrichment of MAPK pathway mutations in advanced cancers and demonstrate that these alterations can promote a switch in pathway dependence from PI3K/AKT to MEK/ERK, lead to resistance to anti-HER2 therapies, and sensitize these cancers to MEK/ERK inhibitors.

## Results

### MAPK pathway alterations in advanced, treatment refractory HER2 + breast cancer

**Fig. 1:**
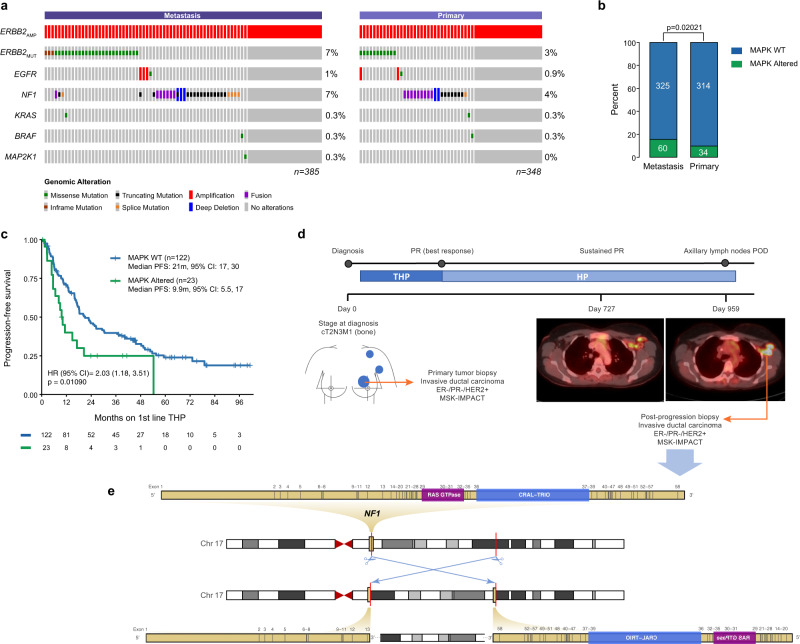
MAPK activating mutations are enriched in metastatic HER2-amplified breast cancers and predict poor response to HER2-targeted therapy. (**a**) Pattern and frequency of MAPK pathway activating mutations (OncoKb annotated) in 733 ERBB2-amplified breast tumors, stratified based on tumor type. (**b**) Frequency of MAPK alterations from (**a**) in metastatic and primary tumor samples, *p* < 0.05 by two-sided Fisher exact test. (**c**) Kaplan-Meier curve displaying progression-free survival of patients receiving first-line anti-HER2 therapy. Analysis was restricted to patients for whom genomic profiling was performed on a tumor specimen prior to starting first-line therapy, *n* = 145. Tumors with functional alterations in MAPK signaling members are shown in green, and tumors without MAPK alterations are shown in blue. *P* < 0.05, two-sided log-rank test. (**d**) The emergence of an NF1- loss of function alteration after exposure to anti-HER2 targeted therapy. This is a case of a 38-year-old female patient with de novo metastatic HER2-positive invasive ductal carcinoma of the left breast, who received first-line treatment with docetaxel, trastuzumab, and pertuzumab (THP), followed by maintenance with trastuzumab and pertuzumab (HP). She experienced a partial response (PR) after 6 months of therapy, which was maintained on HP for 31.5 months (959 days) when she experienced an isolated progression on the left axillary lymph nodes. Fused PET/CT scan axial images of PET Scan on HP and at the time of progression have been shown. Samples of the primary breast tumor and samples of progressing axillary lymph nodes, collected before and after exposure to anti-HER2 therapy, have been sequenced. (**e**) An NF1intragenic inversion (c.1527 + 970:NF1_chr17:g.57371683inv) was detected on left axillary lymph node biopsy but not in the pre-treatment tumor. This rearrangement extends 27 megabases, bisecting NF1 and resulting in inversion of exons 14-58 of NF1; this is predicted to result in loss of function due to involvement and complete inversion of the RAS GTPase domain. Abbreviations: IDC: invasive ductal carcinoma; ER: estrogen receptor: PR: progesterone receptor; POD: progression of disease; PR: partial response; PET: positron emission tomography; CT: computed tomography.

To interrogate the signaling mechanisms underlying resistance to HER2-targeted therapies, we performed genomic sequencing analysis on a cohort of 733 *ERBB2*-amplified primary and metastatic breast tumors (Fig. S1a). As expected, we identified concurrent mutations in the PI3K/AKT pathway including in *PIK3CA* (30%), *PTEN* (2.6%), and *AKT1* (0.3%)^18^. We also found a significant enrichment of mutations known to activate RAS-MAPK signaling (Figs. 1a, S1b–d) in metastatic samples as compared to primary tumors (*p* = 0.020) (Fig. 1b). These alterations included genetic loss of the RAS-GAP *NF1* (8% of metastatic tumors) and activating mutations in *ERBB2* (7% of metastatic tumors). Enrichment analysis of the PI3K/AKT pathway in metastatic samples did not reach statistical significance (*p* = 0.07; OR 1.37 [0.97−1.84]). There were no positive association or mutual exclusivity noted between the PI3K/AKT and MAPK pathways (*p* = 0.42; OR 1.20 [0.74–1.92].

Given the known effect of *PIK3CA* mutation upon response to anti-HER2 therapy^15–18^, we investigated the therapeutic consequences of the RAS-MAPK pathway alterations identified in this cohort. In an analysis of 145 patients with *ERBB2*-amplified tumors sequenced prior to commencement of first-line anti-HER2 therapy, we found a significant (*p* = 0.01) reduction in the progression-free survival (PFS) of patients with MAPK-altered cancers (Fig. 1c). Patients with *ERBB2*-amplified cancers without mutations in MAPK pathway components had a median PFS on first line HER2-targeted therapy of 21 months (95% Confidence Interval [CI]: 17, 30 months), compared to 9.9 months for MAPK-altered patients (95% CI: 5.5, 17 months), suggesting that activation of MAPK signaling limits the efficacy of anti-HER2 agents and contributes to poor patient outcomes (Hazard Ratio [HR]: 2.03, 95% CI: 1.18, 3.51; multivariate *p* = 0.011, univariate log-rank *p* = 0.023). These results remained unchanged after further adjustment of the models for *PIK3CA*, *AKT1,* and *PTEN* alterations known to result in resistance to anti-HER2 therapy (HR: 2.25; 95% CI: 1.29, 3.93; multivariate *p* = 0.0043), indicating that this association is independent of the alterations involving the PI3K pathway. This is further evidenced by the examination of the mutational profile of paired patient pre-treatment and post-progression tumor samples. In a representative case highlighted in Fig. 1d, a patient with de novo metastatic HER2 + invasive ductal carcinoma experienced lymph node progression on anti-HER2 therapy after an initial response. Sequencing of the lymph node sample revealed an *NF1* intragenic inversion that was not present in the pre-treatment tumor. This inversion is predicted to result in loss of function of *NF1* due to involvement of the RAS GTPase domain (Fig. 1e) and thus points to a role for RAS activation in anti-HER2 therapy-resistant disease.

### MAPK pathway activation promotes resistance to anti-HER2 therapies

**Fig. 2:**
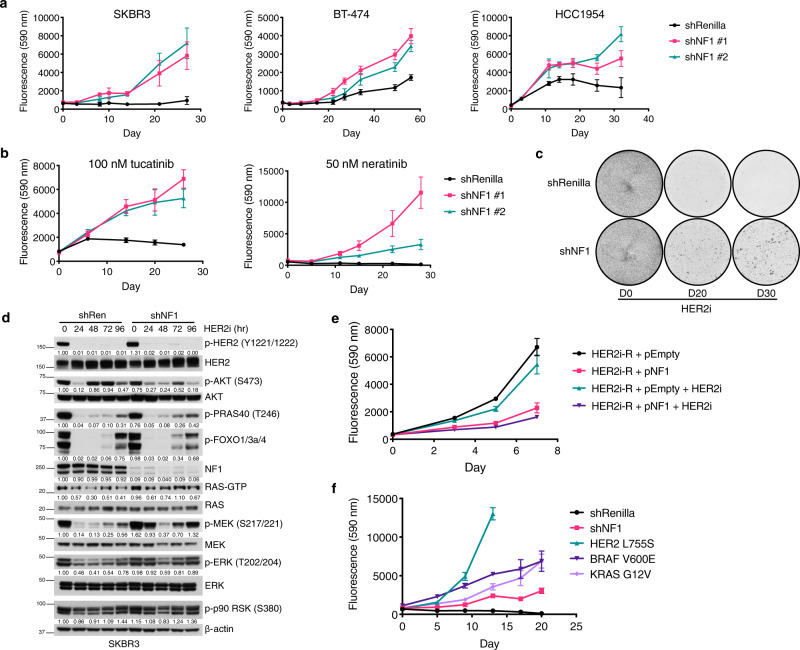
MAPK activating mutations promote resistance to HER2 inhibition. (**a**) Proliferation of shRenilla control and shNF1 HER2 + breast cancer cell lines exposed to 500 nM (SKBR3, MDA-MB-361, BT-474) or 2 uM (HCC1954) lapatinib. shNF1 #1 and #2 represent unique short hairpin sequences targeting NF1. Data are means + /- SD of six biological replicates. (**b**) Proliferation of shRenilla and shNF1 expressing SKBR3 cells exposed to 50 nM neratinib or 100 nM tucatinib. Data are means of 6 biological replicates ± SD. (**c**) Crystal violet staining of shRenilla and shNF1 SKBR3 cells exposed to 500 nM lapatinib (HER2i) over 30 days. (**d**) Immunoblots of indicated hosphor (*p*) and total proteins in shRen and shNF1 SKBR3 cells were treated with 500 nM lapatinib and collected at specified times. Densitometric quantification values are provided below immunoblots. Phospho-signal intensities were normalized to respective total protein signals. Data are representative of 3 biological replicates. (**e**) Proliferation of lapatinib resistant (HER2i-R) shNF1 SKBR3 cells transduced with a doxycycline (dox)-inducible NF1 or empty control vector, treated with dox ± 500 nM lapatinib. Data are means of 6 biological replicates, ± SD. (**f**) Proliferation of SKBR3 cells transduced with vectors constitutively expressing HER2 L755S, BRAF V600E, KRAS G12V, or shRNAs against NF1 continuously treated with 500 nM lapatinib. Data points are means ± SD, *n* = 6 biological replicates. Source data for all assays are provided as a Source Data file.

Our results identify an enrichment of mutations that promote RAS activation among metastatic HER2 + breast cancers that respond poorly to anti-HER2 therapy, with biallelic *NF1* loss the most common alteration. To determine whether *NF1* loss limits the efficacy of anti-HER2 therapy, we depleted NF1 expression in a panel of HER2 + breast cancer cell lines using either short hairpin (sh) RNAs or CRISPR/Cas9 (Fig. S2a, b). We then examined the impact *NF1* loss has on response to the FDA-approved HER2 kinase inhibitors lapatinib, neratinib, and tucatinib. *NF1* deficient HER2 + cell lines exhibited resistance to all three agents (Figs. 2a-c, S2d, e) in both metabolic and colony formation assays of cell growth compared to control lines. Resistance to the growth inhibitory effects of HER2 kinase inhibition manifested as early as 3-5 days (Fig. S2c) and was more evident with prolonged treatment (>2 weeks, Fig. 2a-c).

Given that RAS activation can induce both the PI3K^24^ and MAPK pathways, we investigated the effect of HER2 inhibition (HER2i) on signaling in these isogenic pairs. Steady-state activation of RAS and MEK/ERK was marginally increased in shNF1 cells compared to shRenilla control (Fig. 2d). Treatment of these cells with 500 nM lapatinib or 100 nM tucatinib led to potent and durable inhibition of HER2 phosphorylation in both shNF1 and shRenilla cells indicative of equivalent target inhibition (Figs. 2d, S2f, g). Interestingly, inhibition of AKT signaling was greater in the shNF1 cells compared to control. By contrast, there was a failure to potently or durably suppress RAS-GTP, phospho-MEK, or phospho-ERK in the shNF1 cells. These data imply that NF1 loss renders the MAPK pathway, but not the AKT pathway, insensitive to the effects of HER2 kinase inhibition in HER2 + breast cancer cells.

To confirm that this observed resistance was specifically mediated by loss of NF1, we first derived stable, anti-HER2 resistant NF1 deficient cell lines by continuous culture in HER2i. We then transduced the shNF1 HER2-inhibitor resistant (HER2i-R) SKBR3 cells with a doxycycline-inducible NF1 expression vector. Re-expression of NF1 (Fig. S2h) inhibited cell proliferation (Fig. 2e) and increased sensitivity to HER2i as indicated by growth (Figs. 2e, S2i) and Rb phosphorylation (Fig. S2h), demonstrating that resistance to HER2i is driven by NF1 loss.

As our sequencing analysis of advanced HER2 + breast tumors identified recurrent mutations in multiple MAPK pathway members (Fig. 1a), we asked whether pathway activation through mutational mechanisms other than *NF1* loss also promotes resistance to HER2i. Expression of the activated mutants HER2 L755S, BRAF V600E, or KRAS G12V in SKBR3 cells all increased proliferation under HER2 kinase inhibitor treatment (lapatinib, tucatinib) as compared to vector control cells (Figs. 2f, S2j). These results reveal that MAPK pathway mutations promote resistance to HER2 inhibition in vitro and may do so through re-activation of MAPK-signaling.

### MAPK activating mutations confer MEK/ERK hypersensitivity

**Fig. 3:**
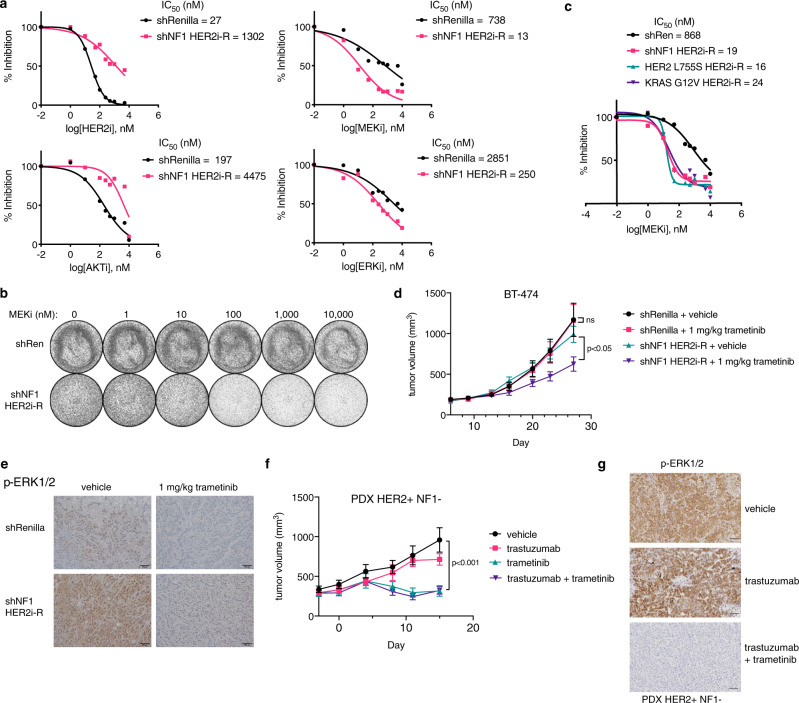
MAPK pathway activating mutants promote MEK dependence. (**a**) Inhibition of proliferation of SKBR3 shRenilla and shNF1 HER2i-R cells by lapatinib (HER2i), MK2206 (AKTi), trametinib (MEKi), and SCH772984 (ERKi), plotted as % inhibition of proliferation after 5 days treatment vs log concentration of drug (nM). Data represent means of 6 biological replicates. (**b**) Crystal violet staining of shRenilla and shNF1 HER2i-R SKBR3 cells exposed to indicated doses of trametinib (MEKi) over 7 days. Data are representative images of 3 biological replicates. (**c**) Inhibition of proliferation of shRen and HER2 inhibitor resistant (HER2i-R) shNF1, HER2 L755S-expressing, and KRAS G12V-expressing SKBR3 cells by trametinib (MEKi). (**d**) Growth of BT-474 shRenilla and shNF1 HER2i-R xenograft tumors treated with vehicle or 1 mg/kg trametinib daily. *n* = 10 mice per group, data are means ± SEM. *P* value = 0.0059 by two-sided student’s *t*-test. (**e**) Immunohistochemistry staining of phosphor-ERK1/2 in BT-474 xenograft tumor sections from (**d**). Images are representative of 4 tumors per treatment group. Scale bars = 100 um (**f**) Growth of patient-derived xenograft harboring ERBB2 amplification and NF1 deletion in mice treated with vehicle, 10 mg/kg trastuzumab bi-weekly, 1 mg/kg trametinib weekly, or the combination. *N* = 5 mice per group bearing two tumors each, data are means ± SEM. *P* value < 0.00103 by two-sided student’s *t*-test. (**g**) Immunohistochemistry staining of phospho-ERK in NF1 null PDX tumors from (**f**), scale bar 100 um. Source data for all assays are provided as a Source Data file.

HER2 + breast cancers are dependent upon PI3K-AKT signaling^4^ and insensitive to MEK/ERK inhibition^3^. However, we observed NF1 loss to specifically render the MAPK pathway insensitive to the effects of HER2 inhibition, raising questions about the ‘driver pathway’ in this context. We therefore assessed the response of HER2 inhibitor-resistant (HER2i-R, Fig. 3a) shNF1 SKBR3 cells to selective inhibitors of either pathway. Compared to shRenilla control cells, shNF1 HER2i-R cells were markedly resistant to AKT inhibition (MK2206 IC_50_ shift from 197 to 4475 nM). Moreover, shNF1 HER2i-R cells were sensitized to MEK (trametinib IC_50_ from 738 to 13 nM) and ERK (IC_50_ from 2851 to 250 nM) inhibition (Fig. 3a, b), indicating a switch in pathway dependence from AKT to MAPK. In contrast, we found that inhibition of MAPK signaling via SHP2 inhibition did not block the proliferation of HER2i-R cells (Fig. S3b), likely due to poor inhibition of phospho-MEK and phospho-ERK (Fig. S3c). More complete inhibition of signaling, via MEKi/ERKi (Fig. S3d), is required to block growth in MAPK activated HER2i-resistant cells. KRAS- and HER2- mutant-expressing cells were also sensitized to MEK inhibition (trametinib IC_50_ shift from 868 nM to 24 nM and 16 nM, respectively) to a degree comparable to NF1 deficient cells (19 nM) (Fig. 3c).

To test whether this striking MEK sensitization was held in vivo, we implanted BT-474 shRenilla and shNF1 HER2i-R xenografts and treated them with vehicle or 1 mg/kg trametinib daily for four weeks (Fig. S3a). While shRen and shNF1 HER2i-R tumors grew at a comparable rate, only the shNF1 tumors exhibited a partial response (37% growth inhibition) to single-agent MEK inhibition while the shRenilla tumors were unaffected (non-significant % change in volume) (Fig. 3d). Immunohistochemistry staining of phospho-ERK indicated elevated MAPK activity in shNF1 HER2i-R tumors that was potently inhibited by single-agent trametinib treatment (Figs. 3e, S3e). To further examine the sensitivities of NF1 deficient tumors in a more clinically relevant context, we treated a HER2 + NF1 null patient-derived xenograft (PDX) model derived from a patient with trastuzumab-refractory breast cancer with trastuzumab, trametinib, or the combination. Tumor growth was unaffected (n.s.) by trastuzumab treatment but potently inhibited by MEK inhibition (70% growth inhibition) (Fig. 3f). ERK phosphorylation was fully ablated in tumors exposed to the MEK inhibitor as compared to trastuzumab (Fig. 3g), correlating with the observed difference in tumor growth. Taken together, our findings indicate RAS activation via NF1 loss promotes a switch in pathway dependence from AKT to MAPK, resulting in resistance to anti-HER2 therapy and MEK sensitivity both in vitro and in vivo.

### Cell cycle regulation underlies HER2-inhibitor resistance and MEK dependence

**Fig. 4:**
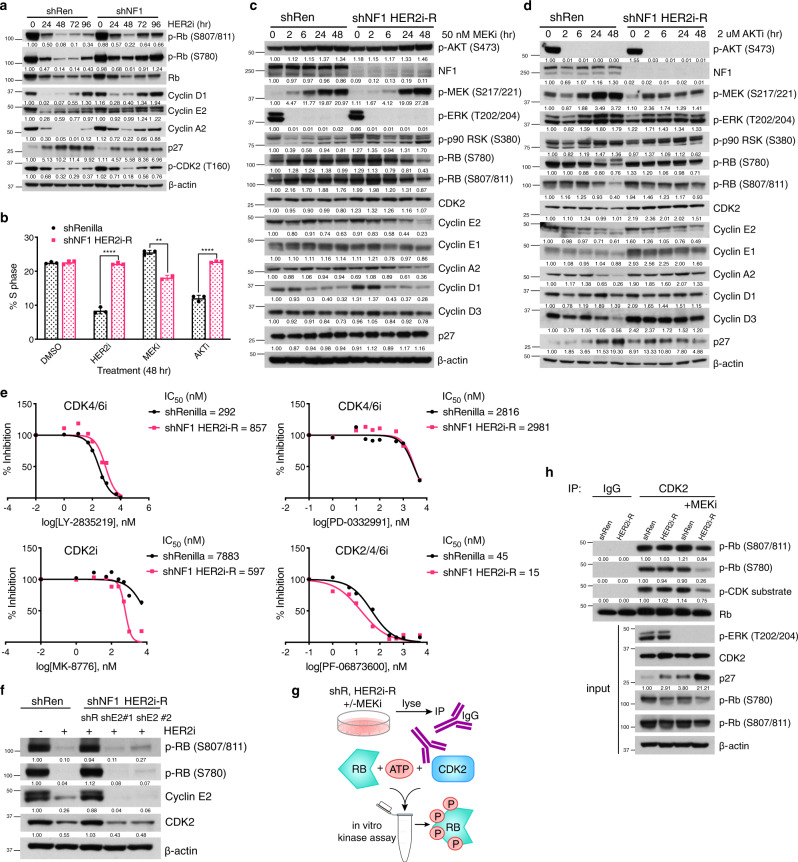
A switch in cell cycle control underlies HER2-inhibitor resistance and MEK dependence of MAPK-activated HER2 + breast cancer cells. (**a**) Immunoblots of indicated cell cycle regulatory proteins in shRenilla control and shNF1 SKBR3 cells treated with 500 nM lapatinib (HER2i) for 0, 24, 48, 72, and 96 h. Images are representative of 3 biological replicates. Quantification normalized to beta-actin levels. (**b**) The cell cycle distribution of SKBR3 shRen control and shNF1 HER2i-R cells treated with DMSO, 500 nM lapatinib (HER2i), 50 nM trametinib (MEKi), or 2 uM MK2206 (AKTi) as measured by fluorescence-activated cell sorting, plotted as % of cells in S phase. Data are means± SD of three independent experiments. (*p* = 1.833 × 10^−5^, 1.03 × 10^−3^, 2.9 × 10^−5^, two-sided student’s *t*-tests). (**c** and **d**) Immunoblots of indicated proteins in cells from (**b**) treated with 50 nM trametinib (MEKi, c) or 2 uM MK2206 (AKTi, d) and collected at 0, 2, 6, 24, and 48 h. Images are representative of 3 biological repeats. (**e**) Inhibition of proliferation of shRenilla and shNF1 HER2i-R SKBR3 cells treated with increasing doses of indicated CDK inhibitors. Data points represent the mean of 6 biological replicates. (**f**) Immunoblots of phospho-Rb, cyclin E2, CDK2, and β-actin in SKBR3 shRen and shNF1 HER2i-R cells transduced with dox-inducible shRNAs against cyclin E2 or renilla, cultured in dox and 500 nM lapatinib for 48 h. (**g**) Graphical overview of CDK2 immunoprecipitation (IP) kinase assay. CDK2 (or IgG control) was immunoprecipitated from shRen control and shNF1 HER2i-R cells treated with DMSO or 50 nM trametinib (MEKi) for 48 h and incubated in an in vitro kinase assay using recombinant Rb1 substrate. (**h**) Western blot analyses of results of the IP kinase assay described in (**g**) and assay input. Source data for all assays are provided as a Source Data file.

HER2 inhibition impedes tumor growth by affecting several hallmarks of cancer including suppression of cell cycle progression^25–27^, induction of apoptosis^28^, and inhibition of cap-dependent translation^29,30^. To determine how NF1 loss drives resistance to HER2 inhibition, we investigated which of these “outputs” were rendered HER2 independent and MEK dependent. Upon HER2i, cleavage of PARP, caspase 3, and caspase 7, as well as inhibition of mTOR activity (substrate phosphorylation) were similar in both control and shNF1 SKBR3 cells (Fig. S4a). In contrast, the inhibition of pRb phosphorylation and G1 cyclin expression by HER2 blockade was weaker and less durable in NF1 deficient cells than in control cells (Figs. 4a, S4a), and was tightly correlated with the observed rebound of MAPK activity (Fig. 2d). We hypothesized that RAS may be driving G1/S phase cell cycle progression to promote resistance to HER2i, and therefore examined the cell cycle distribution of HER2i-resistant cells treated with HER2, AKT, or MEK inhibitors using FACS. The percentage of shNF1 HER2i-R cells in the S-phase was unaffected by either HER2 or AKT inhibition but was reduced from 23% to 18% upon MEK inhibition (Fig. 4b). The inverse was true of shRen control cells where the percent of cells in S-phase was reduced by HER2i (from 22% to 9%)/AKTi (to 12%) and unchanged by MEKi (26%), implying that NF1 loss shifts regulation of the G1/S checkpoint from the AKT to MEK pathway.

To confirm these findings, we assessed protein levels of cell cycle regulators over the course of treatment with 50 nM MEKi (Fig. 4c) or 2 uM AKTi (Fig. 4d). MEK inhibition of shNF1 HER2i-R cells resulted in diminished pRb phosphorylation, decreased cyclin E2, A2, and D1 expression, and induction of p27 levels. In control cells, these proteins, with the exception of cyclin D1, were unaffected by MEK inhibition. Conversely, AKT inhibition reduced Rb phosphorylation and cyclin A/E levels and induced p27 expression in control cells but not in shNF1 cells. Thus, control of the G1- to S-phase cell cycle transition switches from AKT to MEK/ERK in NF1 deficient HER2 + cells, resulting in MAPK dependence.

As our data implicate a rewiring of the G1/S checkpoint in promoting resistance to HER2i, we sought to identify the CDK responsible by first evaluating the consequences of selective inhibition of CDK2 and CDK4/6 in these models. We found shNF1 HER2i-R cells to be resistant (equally or more so than control cells) to CDK4/6 inhibition, but more sensitive to CDK2 or CDK2/4/6 inhibition (MK-8776 IC_50_ shift from 7883 to 597 nM, PF-06873600 shift from 45 to 15 nM) (Fig. 4e). This was also true of cells expressing HER2 L755S, which were sensitive to CDK2, not CDK4/6 inhibition (Fig. S4b). Genetic ablation of CDK2 activity via shRNA knockdown of cyclin E2 expression in shNF1 HER2i-R cells also reduced Rb phosphorylation to levels comparable to control cells treated with lapatinib (Fig. 4f), again pointing to the role of cyclin E/CDK2 in mediating resistance to HER2 inhibition.

To further establish the impact of MAPK activation upon CDK2/Cyclin E, we examined CDK2 kinase activity. We immunoprecipitated CDK2 (Fig. S4c) from control and resistant (shNF1 HER2i-R) cells treated with a MEK inhibitor or DMSO and performed in vitro kinase assays using recombinant pRb1 substrate (Fig. 4g). CDK2 activity in the resistant cells, as indicated by phosphorylation of pRb1, was substantially reduced by MEK inhibitor treatment (80% reduction, Fig. 4h) and correlated with a marked (6-fold) induction of p27 abundance (Fig. 4h). By contrast, CDK2 activity in the control cells was unaffected (± 10%) implying a shift in CDK2 regulation as a result of MAPK activation in the resistant cells. These data demonstrate that MAPK activation in HER2 + breast cancers promotes a switch in cell cycle control from AKT to MAPK that drives resistance to HER2-targeted agents but also enforces a strong and targetable dependence on MEK/ERK signaling.

## Discussion

In this study, we utilize metastatic tumor sequencing analyses and preclinical models to demonstrate that MAPK-activating mutations comprise a unique mode of acquired resistance to HER2-targeted therapies. While previous studies have reported the uncommon presence of MAPK pathway alterations in HER2 + breast cancer^31–35^, we utilize a cohort of both primary and metastatic tumors to demonstrate that these mutations are enriched in advanced cancers with prior exposure to HER2-targeted therapies. In this context, the alterations predict poorer outcomes on treatment suggesting that they may be directly contributing to anti-HER2 therapy resistance but also raising the possibility that they may be vulnerable to MAPK pathway targeted therapeutics.

Given the profound dependence of HER2 + breast cancers on PI3K/AKT signaling and the previous reports implicating PI3K activation in trastuzumab/HER2i resistance, we anticipated RAS activation in this context would also serve to reactivate PI3K and promote further dependence upon AKT. Instead, we found that MAPK pathway activating mutations switched HER2+ breast cancer models from AKT- to MEK/ERK-dependent both in vitro and in vivo. This suggests that while HER2 amplification and subsequent ERK activation does not engender MEK dependence, “second hits” in the pathway may be sufficient to overcome feedback suppression of signaling and elicit “output” from the MAPK pathway sufficient to drive tumor growth. These findings are in accordance with studies done in melanoma and non-small cell lung cancer which define the feedback networks responsible for limiting MEK dependence in these cancers and the pathway mutations capable of disabling these networks^3,36–40^. In HER2-amplified breast cancer, it has been reported that *PTEN* deletion causing MAPK hyperactivation is one such mutation^41^, and here we demonstrate that *NF1* loss, *ERBB2,* and *KRAS* activating mutations, among others, also suffice. Whether this downstream activation of MAPK signaling can elaborate additional phenotypes such as metastatic progression is unknown and will be important to address in future studies. While the rewiring of signaling downstream of HER2 upon mutational activation of MAPK signaling does result in resistance to HER2-targeted therapies, it also represents a targetable vulnerability in refractory HER2 + breast cancer. Patients with MAPK pathway-altered cancers who have progressed on standard therapies may benefit from the addition of a MEK/ERK inhibitor to their treatment regimen.

We interrogated the mechanism underlying the acquired MEK sensitivity of our MAPK-activated models and found control of the G1/S checkpoint to be shifted from AKT to MEK control. Given the success of CDK4/6 inhibitors in treating estrogen receptor (ER)-positive breast cancers^42^, there has been increasing interest in targeting the G1/S checkpoint in HER2 + tumors. Preclinical studies establishing cyclin D/CDK4 as a key effector of AKT signaling and as a potential driver of HER2i resistance^23^ support this notion, and a number of clinical trials combining CDK4/6 inhibitors with anti-HER2 therapy are underway^43–45^. Our results, however, point to CDK2 as the CDK mediating MEK-dependent cell cycle progression and HER2i resistance. Consistent with findings in other cancers^46^, MAPK activation promotes insensitivity to CDK4/6 inhibition, whereas our models acquire marked sensitivity to CDK2 inhibition. Cyclin E amplification (and the resulting elevated CDK2 activity) is a well-described if uncommon potential mode of acquired resistance to trastuzumab and CDK2 inhibition has been proposed as a method of countering this resistance^22^. Our findings lend credence to this proposition as we establish CDK2 as the primary effector of MAPK in HER2-inhibitor-resistant breast cancer cells. Early results from trials in HER2 + breast cancers suggest CDK4/6 inhibition may have limited activity in ER-negative^47^ or heavily pre-treated^48^ tumors. We speculate that CDK2 may be more active in treatment-refractory HER2-amplified cases, and patients may derive greater benefit from CDK2 inhibition than CDK4/6i.

Beyond the implications for the treatment of HER2 + breast cancers, our results emphasize the plasticity of growth factor signaling networks. Mutations acquired under the selective pressure of targeted therapy are capable of profoundly shifting not just the driver, but also the downstream pathway dependencies of tumors, disabling feedback suppressive mechanisms, and rewiring signaling input to effector pathways like the cell cycle. While preclinical efforts are useful in delineating the mechanistic consequences of acquired mutations, extensive sequencing of tumors pre- and post-treatment is required to fully comprehend the scope of these signaling phenomena. Identification of such modes of resistance should enable development of effective combinations of the driver and the downstream pathways that can yield more durable antitumor responses in the clinic.

## Methods

### Human subjects

A total of 733 breast tumor specimens from 664 patients with HER2 + metastatic breast cancer who underwent prospective clinical genomic profiling between April 2014 and February 2021. This study was approved by the Memorial Sloan Kettering Cancer Center Institutional Review Board (IRB) and all patients provided written informed consent for tumor sequencing and review of patient medical records for detailed demographic, pathologic, and treatment information (NCT01775072). Detailed treatment history data were obtained for each patient and included all lines of systemic therapy from the time of diagnosis of invasive carcinoma to the study data lock in February 2021. The exact regimen as well as the dates of start and stop of therapy were recorded for each treatment line.

To assess the effect of MAPK alterations on response to anti-HER2 therapy, we identified a subgroup of patients who received first line standard of care taxanes plus trastuzumab and pertuzumab (THP) and underwent tumor sequencing on a sample that was collected prior to start of therapy. The final cohort suitable for survival analysis included 145 patients.

### Prospective sequencing and analysis

For all 733 patients, tumor and patient-matched normal DNA samples were extracted respectively form formalin-fixed paraffin-embedded (FFPE) tumor biopsy samples and matched nucleated cells from peripheral blood. All specimens underwent next-generation sequencing in a CLIA-certified laboratory using MSK-IMPACT, a hybridization capture-based next-generation sequencing assay, which analyzes all protein-coding exons and selected intronic and regulatory regions of 341 to 505 cancer-associated genes, all as previously described^34,49,50^. The average sequencing coverage across all tumors was 663X. Somatic mutations, DNA copy number alterations, and structural rearrangements were identified as previously described^49^ and all mutations were manually reviewed. In addition to the gene-level amplification and deletion calls generated by the clinical laboratory pipeline, genome-wide total and allele-specific DNA copy number were determined using the FACETS^51^. Purity, average ploidy, and allele-specific integer-copy number for each segment were then determined by maximum likelihood. Loss of heterozygosity (LOH) was determined based on the allele-specific integer-copy numbers for tumor suppressors.

### MAPK alterations

For each gene in the genes involved in the MAPK pathway (*ERBB2*, *EGFR*, *NF1*, *KRAS*, *BRAF*, and *MAP2K)* oncogenic relevance of specific variants and copy number changes was assessed using the latest versions of the OncoKB (www.oncokb.org)^52^ and only somatic mutations that were annotated to be pathogenic were included in the analysis.

### Statistical analysis

We determined the association between genomic alterations and progression-free survival (PFDS) on the first line THP from the time of the start of therapy to the time of disease progression or patient death. We used univariate and multivariate Cox proportional hazard models to determine the association between genomic alterations involving the MAPK pathway and PFS. The multivariate models were further for sample type (metastatic vs primary tumor), tumor hormone receptor status (positive vs negative), and whether *ERBB2* amplification was identified by MSK-IMPACT. The models were also adjusted for the presence or absence of pathogenic alterations involving the PI3k pathway (i.e. *PIK3CA, AKT1,* and *PTEN*). We tested the proportionality assumption of the Cox regression model through time-dependency analysis of selected genetic alterations (cox.zph function of the R package survival). We rejected the null hypotheses with a two-sided α = 0.05. The association between categorical variables were assessed using the chi-square test or Fisher Exact test as appropriate.

### Cell lines and reagents

All cell lines used in this study were obtained from ATCC and maintained in a humidified atmosphere with 5% CO_2_ at 37 °C. SKBR3, BT-474, MDA-MB-361, and HCC1954 cell lines were maintained in DMEM/F12 supplemented with 10% FBS, 4 mM glutamine, 100 U/mL penicillin, and 100 ug/mL streptomycin. 293 T cells were maintained in DMEM containing the aforementioned supplements. All cell lines tested negative for mycoplasma. Lapatinib resistant (HER2i-R) cells were established via continuous culture in 500 nM lapatinib. Lapatinib, neratinib (HKI272), trametinib (GSK1120212), MK8776, SCH772984, and PD0332991 were purchased from Selleck Chemicals. MK2206 was obtained from Merck. LY2835219 was provided by Eli Lilly. PF06873600 was purchased from ProbeChem. All compounds were dissolved in DMSO for in vitro studies.

### Plasmids and viral transduction

All short hairpin RNA sequences and expression vectors were gifts from Johannes Zuber^53^. shNF1 #1 (TATATCATGAACATCAACATTG) and shNF1 #2 (TATATCATGAACATCAACATTG) were cloned into SGEP (addgene #111170). shCCNE2 #1 (TAAAATAGTAGTGAGGCCGCTT) and shCCNE2 #2 (TATCACTTTGACACTGTCCTTA) were cloned into LT3REVIN. RTTA expression was achieved via pLenti CMV rtTA3 Blast (addgene #26429). Single guide RNAs against NF1 for CRISPR/Cas9 knockout were designed using crispr.mit.edu (discontinued). sgNF1 #1 GGTCAGCCGCTTCGACGAGC and sgNF1 #2 CGCGCACAGGCCGGTGGAAT were cloned into lentiCRISPR v2 (addgene #52961) with lentiCRISPR - CNTRL sgRNA (addgene #70662) used as a control. For inducible expression of NF1, gateway entry clone R777-E140 Hs.NF1-nostop (addgene #70424, resistant to sh and sgRNAs) was introduced into pInducer20 (addgene #44012). Stable overexpression of cyclin E2 was achieved by cloning the insert from pcDNA3-HA-cyclin E2 (addgene #19935) into pLenti CMV Puro DEST (addgene #17452). pLX302 and pLX302-HER2 L755S expression vectors were gifts from Maurizio Scaltriti. Kras (G12V)-pcw107 (addgene #64602) and pHAGE-BRAF-V600E (addgene #116204) were utilized for mutant KRAS and BRAF expression, respectively. To transduce target HER2 + cell lines, the above lentiviral vectors were transfected into 293 T cells along with VSVG and psPAX2 packaging vectors. Viral supernatant was collected, filtered, and applied to target cells (plus polybrene) prior to selection with the appropriate antibiotic.

### Proliferation assays

For resazurin-based proliferation assays, 500-2,000 cells were plated per well of a 96-well plate with six replicates per condition. After 24 h, cells were treated with inhibitors (Day 0). For long-term culture (>10 days), media (plus inhibitors) was changed weekly. Plates were measured as indicated in figures or for IC_50_ calculations, on day 5, by adding 25 uL resazurin (R&D Systems) to each well and incubating plates at 37 °C for 4 h. Fluorescence (560 nm excitation, 590 nm emission) was read on a SpectraMax M5 plate reader, normalized to media blank. Growth and dose inhibition curves were plotted and analyzed using Graphpad Prism 8. Half maximal inhibitory concentrations (IC_50_) were determined by nonlinear regression analysis of plots of percentage growth inhibition vs log inhibitor concentration. For crystal violet assays, 5 × 10^5^ cells were plated in 6 well plates in triplicate per condition, treated with HER2i, and stained and imaged at time points indicated in figure legends.

### Immunoblotting

Cell lysates were prepared on ice by washing cells once with PBS, resuspending in 1X cell lysis buffer (Cell Signaling Technology #9803) supplemented with Halt protease inhibitor cocktail (Thermo Scientific #78430), and sonicating for 30 s. Lysates were cleared by centrifugation (maximum speed, 10 m) and protein concentration determined by BCA assay (Pierce). 20-60 ug of lysate was loaded on to 4-12% Bis-Tris gels (NuPAGE, Invitrogen) for electrophoresis and immunoblotting. The following antibodies were purchased from Cell Signaling Technology and utilized at 1:5000 dilution: p-AKT (S473) (#4060), AKT (#4691), β-actin (#4970), ERK (#4695), p-ERK (T202/204) (#4370), HER2 (#4290), p-HER2 (Y1221/1222) (#2243), p-PRAS40 (T246) (#13175), p-Rb (S780) (#8180), p-Rb (S807/811) (#8516), p27 (#3686), Rb (#9309), p-S6 (S240/244) (#5364), p-EGFR (Y1068) (#3777). The following antibodies were purchased from Cell signaling Technology and utilized at 1:1000 dilution: CDK2 (#2546), Cyclin A2 (#4656), Cyclin D1 (#55506), Cyclin D3 (#2936), Cyclin E1 (#4129), Cyclin E2 (#4132), E2F1 (#3742), HA (#2367), MEK (#4694),, p-CDK substrate (#9477), p-CDK2 (T160) (#2561),, p-MEK (S217/221) (#9154), p-p90 RSK (S380) (#9341), p-FOXO1/3a/4 (#2599), p-CRAF (S338) (#9427), p-p70 S6K (T389) (#9234), p-4EBP1 (S65) (#9456) (T37/46) (#2855), Cleaved caspase 3 (#9664), Cleaved caspase 7 (#8438), Cleaved PARP (#5625). NF1 antibody was purchased from Abcam (#ab17963) and used at 1:1000 dilution. Active RAS was detected using Thermo Scientific kit #16117. Uncropped immunoblot scans are provided in the Source Data file.

### Xenograft models and in vivo experiments

Animal studies were performed in the MSKCC animal facility in compliance with institutional guidelines under an IACUC approved protocol (MSKCC No. 12-10-016). Health checks were performed daily. To establish BT-474 xenografts, sustained release 0.72 mg 17β-estradiol pellets were implanted subcutaneously into the flanks of six- to eight-week-old athymic female mice at least 3 days prior to cell injection. 10 million BT-474 cells were suspended in Matrigel and injected subcutaneously. Treatment with vehicle (0.5% HPMC, 0.2% Tween80 in H2O pH 8.0) or trametinib was initiated when tumors reached 175 mm3. Animals were euthanized at treatment end, or upon observation of excessive tumor burden or other welfare concerns.

PDX (ER^-^/PR^-^/HER2^3+^) comes from a primary tumor and has been established at VHIO following institutional guidelines. The IRBs at Vall d’Hebron Hospital provided approval for this study in accordance with the Declaration of Helsinki. Written informed consent was obtained from all patients who provided tissue samples. Fragments of patient samples were implanted into the fat pad of NOD. CB17-Prkdcscid [nonobese diabetic (NOD)/severe combined immunodeficient (SCID)] (#SM-NOD-5S-F, Janvier) mice, and and 17b-estradiol (1 mM) (#E8875-1G, Sigma-Aldrich) was added to drinking water. In the experiment, once tumor size reached 200-300 mm3, animals were treated either with vehicle, trastuzumab (10 mg/ml), trametinib (1 mg/kg), or both trastuzumab and trametinib. Tumor xenografts were measured with calipers two times per week, and tumor volume was determined using the following formula: (length x width^2^) x (pi/6).

### Immunohistochemistry

Immunohistochemistry was performed on formalin-fixed paraffin-embedded tumor specimens from cell-derived xenografts. A standard multimer/diaminobenzidine (DAB) detection protocol was performed on Ventana BenchMark ULTRA Automated stainer as previously described^54^, with appropriate negative and positive controls. Phospho-Erk1/2 antibody (Cell Signaling Technology #9101, 1:200 dilution) was used. Images were taken under Leica DMi8 microscope.

### In vitro kinase assays

Cells were washed once with ice-cold PBS, suspended in 1X cell lysis buffer (Cell Signaling #9803) supplemented with protease inhibitor cocktail, and incubated on ice for 5 m followed by sonication for 5 s three times. Lysates were cleared and protein concentration quantified as described above. 500 ug lysate was incubated rotating overnight at 4 °C with CDK2 antibody (CST #2546, 1:50 dilution) or IgG control (CST #3900, concentration matched). The following day, 25 uL ChIP-grade protein A/G magnetic beads (Pierce #26162) per sample were pre-washed twice with cell lysis buffer. Immunocomplexes were transferred to the tubes containing the bead pellets and incubated with rotation at room temperature for 20 m. Beads were then pelleted and washed three times with 1X cell lysis buffer and two times with 1X kinase buffer (CST #9802) on ice. After the final wash, pellets were resuspended in 40 uL of kinase buffer supplemented with 200 uM ATP and 0.25 ug recombinant human Rb substrate (Abcam #ab56270). Kinase reactions were carried out for 30 m at 30 °C and terminated with the addition of sample buffer. Supernatants containing phosphorylated substrate were transferred to new tubes for western blot analysis.

### Reporting summary

Further information on research design is available in the Nature Research Reporting Summary linked to this article.

## Supplementary information

Supplementary Information

Reporting Summary

## Data Availability

The genomic sequencing data generated in this study underlying Figs. 1 and S1 are publicly available via the cBioPortal for cancer genomics (https://cbioportal.org/study/summary?id=brca_mapk_hp_msk_2021). The raw data is available under restricted access, access can be obtained by contacting the corresponding author. The remaining data are available within the Article, Supplementary Information, or Source Data File. Source data are provided with this paper.

## Source data

Source Data
