# Supplementary material for: HER2 + breast cancers evade anti-HER2 therapy via a switch in driver pathway
Source: Nat Commun. 2021 Nov 18;12:6667. doi: 10.1038/s41467-021-27093-y (PMC8602441; doi:10.1038/s41467-021-27093-y)
Supplement: Supplementary file 1 — Supplementary Information [file 41467_2021_27093_MOESM1_ESM.pdf]

Supplementary Information:

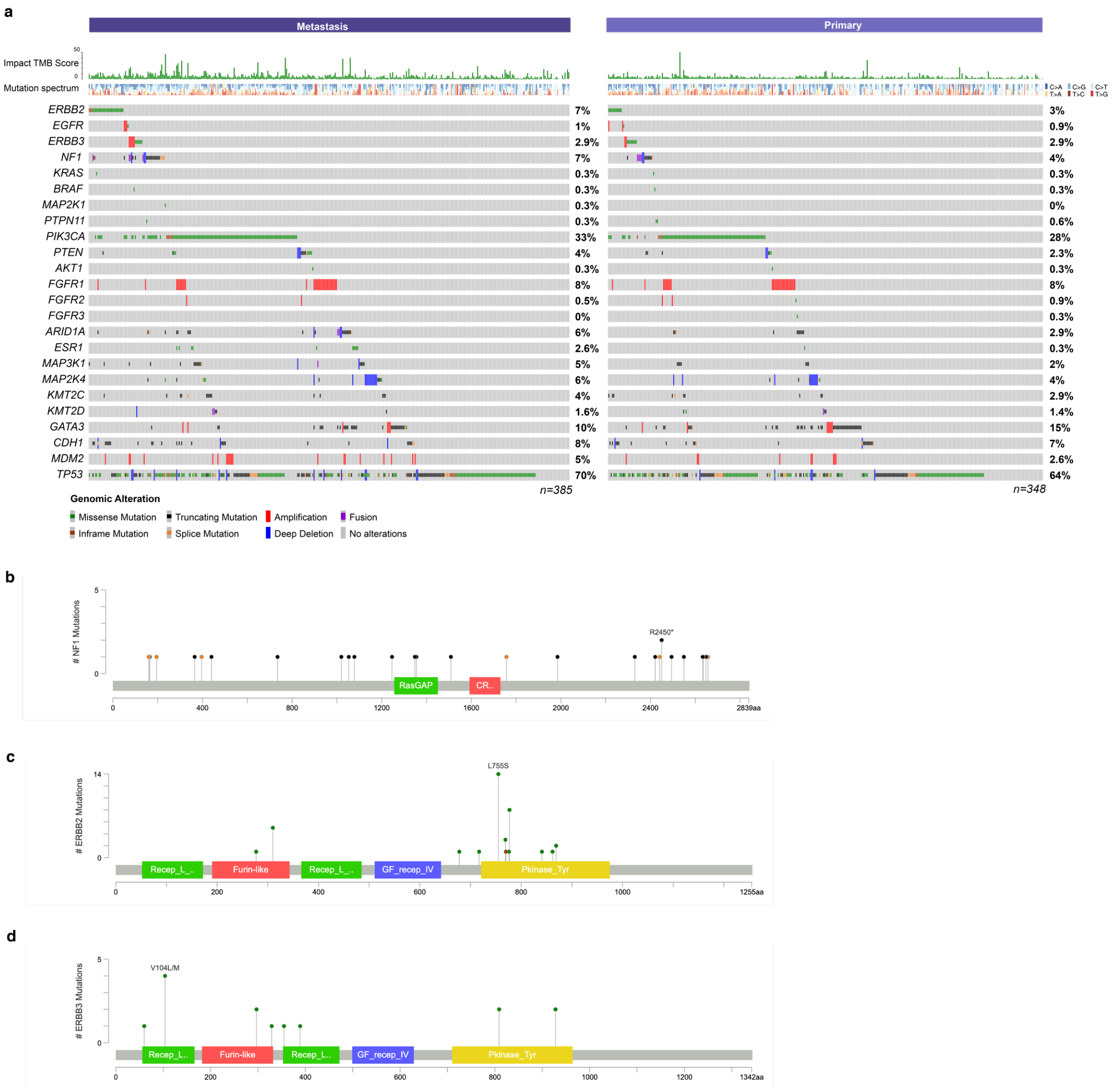

Supplementary Figure 1. Pattern and frequency of mutated genes in ERBB2-amplified breast tumor cohort. **(a)** Oncoprint of frequently mutated genes in 733 ERBB2-amplified breast tumors, stratified based on tumor type. **(b-d)** Lolliplots depicting spectra of *NF1* (b), *ERBB2* (c), and *ERBB3* (d) somatic mutations identified in study cohort.

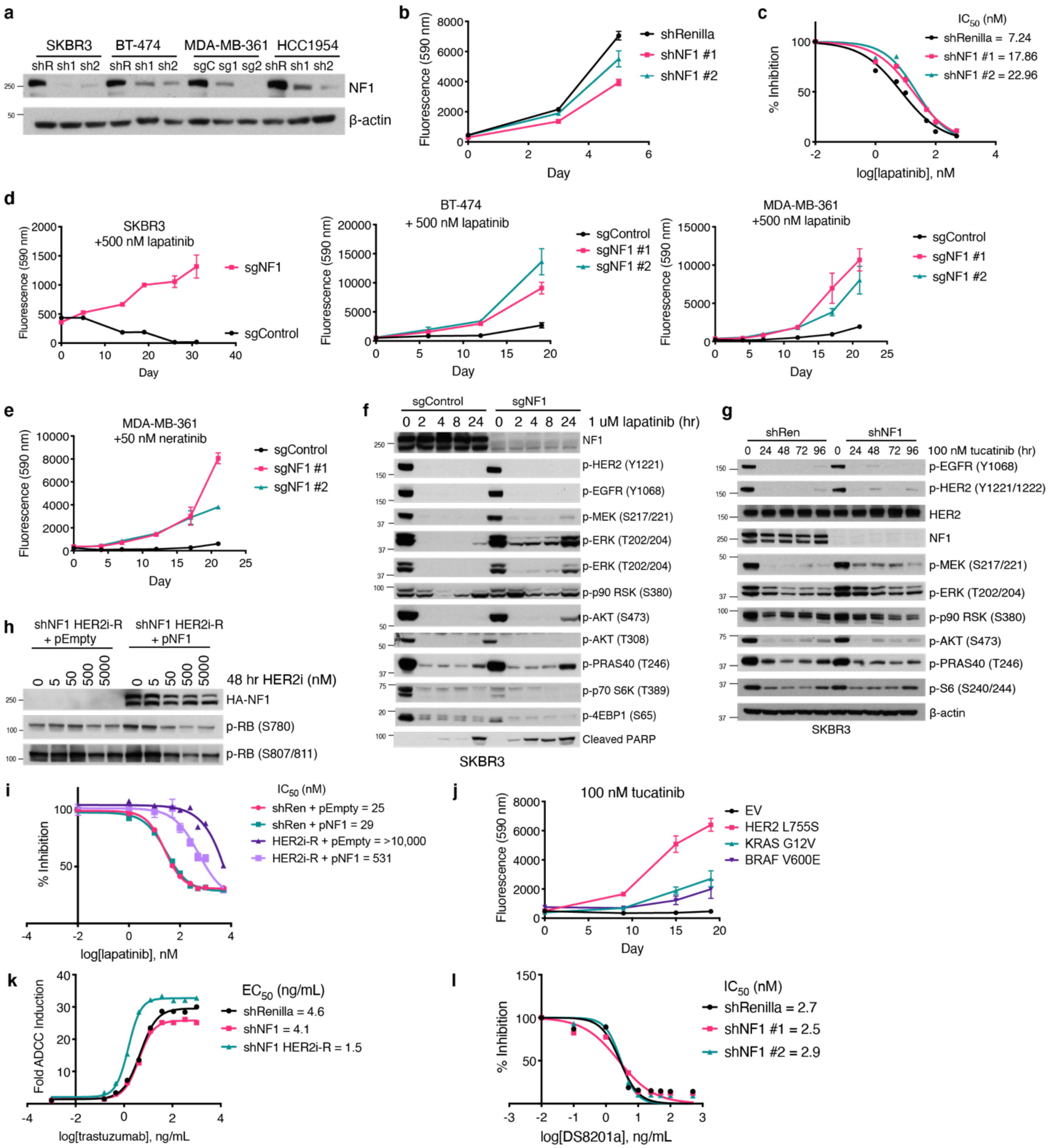

Supplementary Figure 2. NF1 loss promotes resistance to HER2 kinase inhibition. **(a)** Immunoblots of NF1 and β-actin in cells harboring shRNAs (SKBR3, BT-474, HCC1954) or sgRNAs (MDA-MB-361) against NF1. Data are representative images of 3 biological repeats. **(b)** Proliferation of shRen and shNF1 SKBR3 cells without drug treatment. Data are means of 6 biological replicates +/- SD. **(c)** Inhibition of proliferation of shRen or shNF1 cells treated with increasing doses of lapatinib for 3 days. Data are means of 6 biological replicates. **(d)** Proliferation of HER2+ cell lines harboring CRISPR/Cas9 knockout of *NF1* utilizing two unique guide RNA sequences targeting different regions of the gene (sgNF1 #1 and #2) or non-targeting control sgRNA (sgControl) exposed to 500 nM lapatinib. Data points are means of 6 biological replicates +/- SD. **(e)** Proliferation of sgControl or CRISPR knockout sgNF1 MDA-MB-361 cells exposed to 50 nM neratinib, mean +/- SD, n=6 biological replicates. **(f)** Immunoblots of indicated

proteins in sgControl or sgNF1 SKBR3 cells from (d) exposed to 1 uM lapatinib for 0, 2, 4, 8, and 24 hours. Position of molecular weight markers (kDa) indicated to left of panels. Data are representative images of 2 biological repeats. **(g)** Immunoblots of indicated proteins in shRenilla and shNF1 SKBR3 cells treated with 100 nM tucatinib and collected at 0, 24, 48, 72, and 96 hours. Representative images of 3 biological repeats. **(h)** Immunoblots of HA and phospho-Rb in lapatinib-resistant (HER2i-R) shNF1 SKBR3 cells transduced with dox-inducible empty or HA-tagged NF1 expression vectors treated with dox and indicated doses of lapatinib for 48 hours. **(i)** Inhibition of proliferation of shRen and shNF1 HER2i-R cells harboring expression vectors from (h) by lapatinib. Data points represent mean of 6 biological replicates +/- SD. **(j)** Proliferation of SKBR3 cells expressing vector control, HER2 L755S, KRAS G12V, or BRAF V600E exposed to 100 nM tucatinib. Data are means of 6 biological replicates +/- SD. **(k)** Induction of ADCC by trastuzumab treatment of shRenilla, shNF1, or shNF1 HER2i-R SKBR3 cells. SKBR3 cells were incubated with ADCC effector reporter cells (Promega) and trastuzumab before measurement of luciferase signal. Fold change in signal intensity is plotted against the logarithmic antibody concentration. Data are means of 6 biological replicates. **(l)** Inhibition of proliferation of shNF1 and control SKBR3 cells exposed by DS-8201a after 5 days. Data are means of 6 biological replicates.

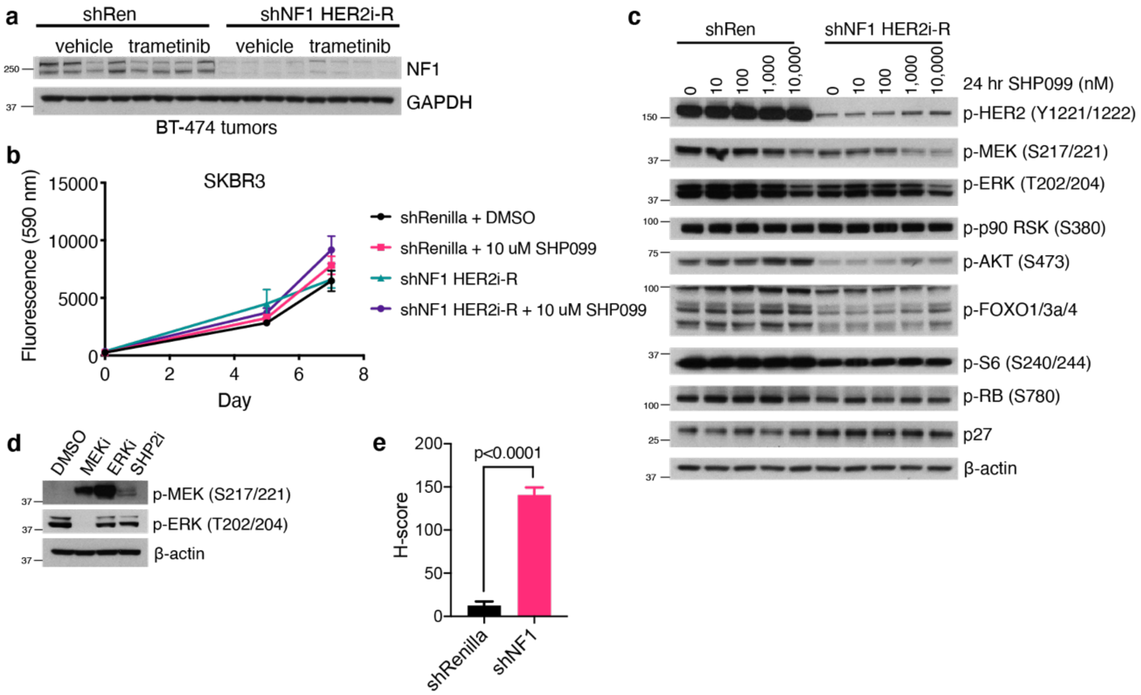

Supplementary Figure 3. MAPK activating mutations sensitize HER2+ breast cancer cells to MEK/ERK inhibition. **(a)** Immunoblots of NF1 and GAPDH in BT-474 shRenilla or shNF1 HER2i-R tumors treated with vehicle or 1 mg/kg trametinib daily. Four tumors are depicted per treatment group. Xenograft experiment was repeated twice. **(b)** Proliferation of SKBR3 shRenilla and shNF1 HER2i-R cells treated with DMSO or 10 uM SHP099. Data are means of 6 biological replicates +/- SD. **(c)** Immunoblots of indicated proteins in cells from (b) treated with 0-10,000 nM SHP099 for 24 hours. Data are representative of 2 biological replicates. **(d)** Immunoblots of phospho-MEK, phospho-ERK, and actin in SKBR3 shNF1 HER2i-R cells treated for 48 hours with 50 nM trametinib (MEKi), 1 uM SCH772984 (ERKi), or 10 uM SHP099 (SHP2i). Data are representative of 3 biological repeats. **(e)** Phospho-ERK IHC H-scores (staining intensity 0, 1, 2, or 3 multiplied by % of positive cells in each staining category) of BT-474 shRenilla and shNF1 HER2i-R xenograft tumors. Data are means of 4 tumors +/- SD, $p<0.001$ by two-tailed student's t-test. Source data for all assays are provided as Source Data file.

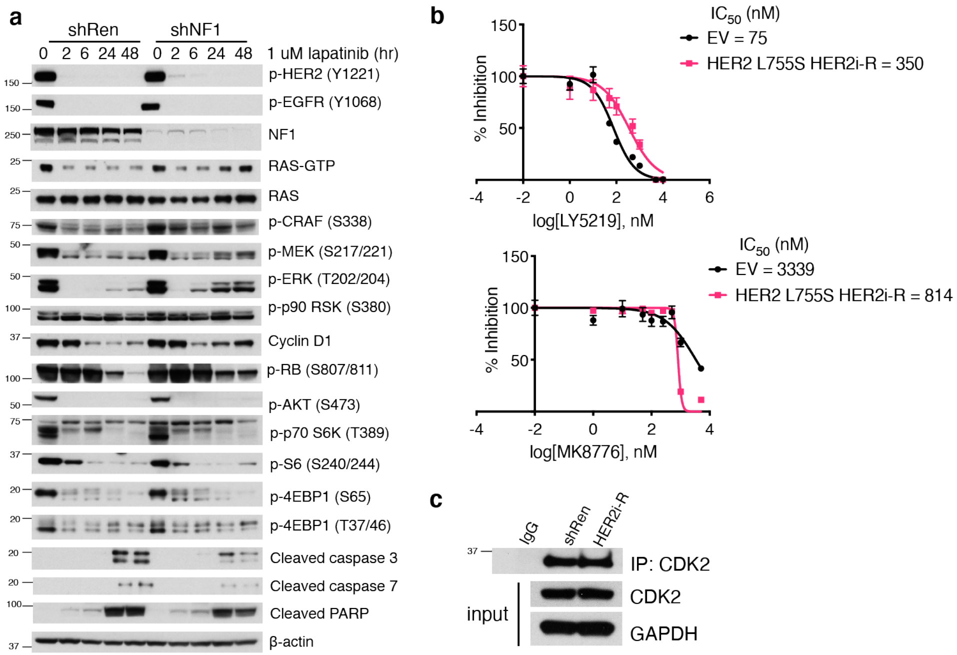

Supplementary Figure 4. A switch in cell cycle control drives HER2 inhibitor resistance and MEK dependence in MAPK-activated HER2+ breast cancer cells. **(a)** Immunoblots of indicated proteins in SKBR3 shRenilla and shNF1 cells treated with 500 nM lapatinib (HER2i) for 0, 2, 6, 24, and 48 hours. Data are representative of 5 biological repeats. **(b)** Inhibition of proliferation of SKBR3 vector control or HER2 L755S mutant-expressing HER2i-R cells by LY5219 or MK8776. Data points are means +/- SD, n=6 replicates. **(c)** Immunoblots of CDK2 and GAPDH in CDK2-immunoprecipated and total input lysates utilized for CDK2 IP kinase assay in Fig. 4g-h. Data are representative of 3 biological replicates.
