# Supplementary material for: HER2 + breast cancers evade anti-HER2 therapy via a switch in driver pathway
Source: Nat Commun. 2021 Nov 18;12:6667. doi: 10.1038/s41467-021-27093-y (PMC8602441; doi:10.1038/s41467-021-27093-y)
Supplement: Supplementary file 2 — Reporting Summary [file 41467_2021_27093_MOESM2_ESM.pdf]

nature research

Corresponding author(s): Sarat Chandarlapaty

Last updated by author(s): Nov 2, 2021

Reporting Summary

Nature Research wishes to improve the reproducibility of the work that we publish. This form provides structure for consistency and transparency in reporting. For further information on Nature Research policies, see our Editorial Policies and the Editorial Policy Checklist.

Statistics

For all statistical analyses, confirm that the following items are present in the figure legend, table legend, main text, or Methods section.

n/a	Confirmed	
☐	☒	The exact sample size (*n*) for each experimental group/condition, given as a discrete number and unit of measurement
☐	☒	A statement on whether measurements were taken from distinct samples or whether the same sample was measured repeatedly
☐	☒	The statistical test(s) used AND whether they are one- or two-sided<br>*Only common tests should be described solely by name; describe more complex techniques in the Methods section.*
☐	☒	A description of all covariates tested
☐	☒	A description of any assumptions or corrections, such as tests of normality and adjustment for multiple comparisons
☐	☒	A full description of the statistical parameters including central tendency (e.g. means) or other basic estimates (e.g. regression coefficient) AND variation (e.g. standard deviation) or associated estimates of uncertainty (e.g. confidence intervals)
☐	☒	For null hypothesis testing, the test statistic (e.g. *F*, *t*, *r*) with confidence intervals, effect sizes, degrees of freedom and *P* value noted<br>*Give P values as exact values whenever suitable.*
☐	☒	For Bayesian analysis, information on the choice of priors and Markov chain Monte Carlo settings
☐	☒	For hierarchical and complex designs, identification of the appropriate level for tests and full reporting of outcomes
☐	☒	Estimates of effect sizes (e.g. Cohen's *d*, Pearson's *r*), indicating how they were calculated

Our web collection on statistics for biologists contains articles on many of the points above.

Software and code

Policy information about availability of computer code

Data collection

Tumor Tissue sequencing:
Tumor sequencing was performed utilizing the FDA-authorized MSK-IMPACT assay (Zehir et al, Nat Med 2017). Briefly, tumor DNA was extracted from formalin-fixed paraffin-embedded (FFPE) biopsy samples and matched normal DNA was extracted from mononuclear cells from peripheral blood. All specimens underwent next-generation sequencing in the MSKCC CLIA-certified laboratory using MSK-IMPACT, an FDA-authorized hybridization capture-based next-generation sequencing assay, which analyzes all protein-coding exons of 341-410 cancer associated genes , as previously described (Zehir et al, Nat Med 2017, Cheng et al, J Mol Diagn 2015). Somatic mutations, DNA copy number alterations, and structural rearrangements were identified as previously described (Cheng et al, J Mol Diagn 2015) and all mutations were manually reviewed.

Base call files generated by Illumina's RTA software (v2.12) were de-multiplexed using bcl2fastq (v2.19) and processed with a custom pipeline for molecule barcode detection, sequencing adapter trimming, and base quality trimming (discarding bases below Q20 at the ends of the reads). Processed reads were then aligned to the human reference genome hg19 using BWA-MEM (arXiv:1303.3997v2) and used to build double-stranded consensus representations of original unique cfDNA molecules using both the inferred molecular barcodes and read start/stop positions. SNVs were detected by comparing read and consensus molecule characteristics to sequencing platform- and position-specific reference error noise profiles determined independently for each position in the panel by sequencing a training set of 62 healthy donors on both the NextSeq 500 and HiSeq 2500. Observed positional SNV error profiles were used to define calling cut-offs for SNV detection with respect to the number and characteristics of variant molecules, which differed by position but were most commonly ≥2 unique molecules, which in an average sample (~5,000 unique molecule coverage), the corresponds to a detection limit of ~0.04% VAF. Indel detection used two methods. For short (<50-70bp) indels, a generative background noise model was constructed to account for PCR artifacts arising frequently in homopolymeric or repetitive contexts, allowing for strand-specific and late PCR errors. Detection was then determined by the likelihood ratio score for observed feature weighted variant molecule support versus background noise distribution. Detection of indels >>50bp relies on secondary analysis of soft-clipped reads using methods described in the fusion section below and is only performed to detect specific genomic events (e.g. MET exon 14-skipping deletions). Reporting thresholds were event-specific as determined by performance in training samples but

were most commonly at least one unique molecule for clinically actionable indels, which in an average sample corresponds to a detection limit of ~0.02% VAF.

To detect CNAs, probe-level unique molecule coverage was normalized for overall unique molecule throughput (the equivalent of library size), probe efficiency, GC content, and signal saturation and summarized at the gene level. CNA determinations were based on training set-established decision thresholds for both absolute copy number deviation from per-sample diploid baseline and deviation from the baseline variation of probe-level normalized signal in the context of background variation within each sample's own diploid baseline. Per-sample relative tumor burden was determined by normalization to the mutational burden expected for tumor type and ctDNA fraction and reported as a z-score.

Data analysis

All analysis was performed using the R environment for statistical computing. A detailed description of the analysis and assumptions made is provided in the Methods section.

The following softwares were used to process and analyze data:
1/ RTA v2.12
2/ bcl2fastq v2.19
3/ BWA-MEM arXiv:1303.3997v2
4/ Samtools v1.3.1
5/ FACETS v0.5.2
6/ CNVkit v0.9.2.dev0
7/ R v3.5.1
8/ Bioconductor version 3.8
9/ ggplot2 v3.2.1
10/ cowplot v1.0
11/ Hmisc v4.2-0
12/ scales v1.0
13/ clinfun v1.0.15
14/ mblm v0.12.1
15/ maftools v1.8.0
16/ lmtest v0.9-36
17/ glmperm v1.0-5
18/ Absolute v1.0.6
19/ vcf2maf v1.6.3
20/ VEP v81

The MSK-IMPACT pipeline used to process and tumor biopsy and matched WBC sequencing together with the full list of dependencies and version information are available from https://github.com/rhshah/IMPACT-Pipeline

Graphpad PRISM

For manuscripts utilizing custom algorithms or software that are central to the research but not yet described in published literature, software must be made available to editors and reviewers. We strongly encourage code deposition in a community repository (e.g. GitHub). See the Nature Research guidelines for submitting code & software for further information.

Data

Policy information about availability of data

All manuscripts must include a data availability statement. This statement should provide the following information, where applicable:

- Accession codes, unique identifiers, or web links for publicly available datasets
- A list of figures that have associated raw data
- A description of any restrictions on data availability

The genomic sequencing data generated in this study underlying Figures 1 and S1 are publicly available via the cBioPortal for cancer genomics (https://cbioportal.org/study/summary?id=brca_mapk_hp_msk_2021). The raw data is available under restricted access, access can be obtained by contacting the corresponding author. The remaining data are available within the Article, Supplementary Information, or Source Data File.

Field-specific reporting

Please select the one below that is the best fit for your research. If you are not sure, read the appropriate sections before making your selection.

☒ Life sciences ☐ Behavioural & social sciences ☐ Ecological, evolutionary & environmental sciences

For a reference copy of the document with all sections, see nature.com/documents/nr-reporting-summary-flat.pdf

Life sciences study design

All studies must disclose on these points even when the disclosure is negative.

Sample size

For mouse studies, sample sizes were determined based on similar studies utilizing breast cancer cell line- and patient-derived xenograft models performed in our laboratory/institution (Will et al., Cancer Discovery 2014, Cocco et al. Science Signaling 2018). We estimated 10

	mice/group would allow for detection of differences in tumor volume >200mm3. Experiments were repeated at least 2-3 times. Sample size of clinical cohort was not pre-determined.
Data exclusions	Four PDX tumors were excluded from analysis due to outlying volume prior to commencement of treatment (+/- 2 SD volume at day -3, pre-established threshhold). No other data were excluded.
Replication	All in vitro and mouse experiments were successfully replicated 2-3 times. Where representative experiments are shown, number of consistent replicates indicated in figure legends. Replication of clinical study was not relevant.
Randomization	Mice were randomly assigned to tumor injection/treatment groups. Clinical studies were not randomized as we were not directly comparing groups. Randomization is not relevant for in vitro cell line experiments.
Blinding	In mouse studies, there was no blinding of the investigator as randomization of animals was done and 2-3 experimental replicates performed. Blinding of clinical study was not relevant as we were not directly comparing groups

Reporting for specific materials, systems and methods

We require information from authors about some types of materials, experimental systems and methods used in many studies. Here, indicate whether each material, system or method listed is relevant to your study. If you are not sure if a list item applies to your research, read the appropriate section before selecting a response.

Materials & experimental systems

n/a	Involved in the study
☐	☒ Antibodies
☐	☒ Eukaryotic cell lines
☒	☐ Palaeontology and archaeology
☐	☒ Animals and other organisms
☐	☒ Human research participants
☐	☒ Clinical data
☒	☐ Dual use research of concern

Methods

n/a	Involved in the study
☒	☐ ChIP-seq
☒	☐ Flow cytometry
☒	☐ MRI-based neuroimaging

Antibodies

Antibodies used	The following antibodies were purchased from Cell Signaling Technology and utilized at 1:5000 dilution: p-AKT (S473) (#4060), AKT (#4691), β-actin (#4970), ERK (#4695), p-ERK (T202/204) (#4370), HER2 (#4290), p-HER2 (Y1221/1222) (#2243), p-PRAS40 (T246) (#13175), p-Rb (S780) (#8180), p-Rb (S807/811) (#8516), p27 (#3686), Rb (#9309), p-S6 (S240/244) (#5364), p-EGFR (Y1068) (#3777). The following antibodies were purchased from Cell signaling Technology and utilized at 1:1000 dilution: CDK2 (#2546), Cyclin A2 (#4656), Cyclin D1 (#55506), Cyclin D3 (#2936), Cyclin E1 (#4129), Cyclin E2 (#4132), E2F1 (#3742), HA (#2367), MEK (#4694), , p-CDK substrate (#9477), p-CDK2 (T160) (#2561), , p-MEK (S217/221) (#9154), p-p90 RSK (S380) (#9341), p-FOXO1/3a/4 (#2599), p-CRAF (S338) (#9427), p-p70 S6K (T389) (#9234), p-4EBP1 (S65) (#9456) (T37/46) (#2855), Cleaved caspase 3 (#9664), Cleaved caspase 7 (#8438), Cleaved PARP (#5625). NF1 antibody was purchased from Abcam (#ab17963) and used at 1:1000 dilution. Active RAS was detected using Thermo Scientific kit #16117. Uncropped immunoblot scans are provided in the Source Data file.
Validation	information in CST, Abcam, and Thermo Scientific certificate of analyses. Cell signaling uses hallmark strategies binary model, ranged expression, orthogonal data, multiple antibodies, heterologous expression, and complementary assays.

Eukaryotic cell lines

Policy information about cell lines

Cell line source(s)	ATCC (SKBR3, BT-474, MDA-MB-361, HCC1954, and 293T
Authentication	STR
Mycoplasma contamination	All lines tested negative for mycoplasma via Lonza myco alert
Commonly misidentified lines (See ICLAC register)	None

Animals and other organisms

Policy information about studies involving animals; ARRIVE guidelines recommended for reporting animal research

Laboratory animals	6-8 week old female athymic, or NOD. CB17-Prkdcscid [nonobese diabetic (NOD)/severe combined immunodeficient (SCID)] (#SM-NOD-5S-F, Janvier)
Wild animals	None

Field-collected samples	None
Ethics oversight	IACUC approved protocol (MSKCC No. 12-10-016), IRBs at Vall d'Hebron Hospital provided approval for this study in accordance with the Declaration of Helsinki

Note that full information on the approval of the study protocol must also be provided in the manuscript.

Human research participants

Policy information about studies involving human research participants

Population characteristics	A total of 733 breast tumor specimens from 664 patients with HER2+ metastatic breast cancer who underwent prospective clinical genomic profiling between April 2014 and February 2021. This study was approved by the Memorial Sloan Kettering Cancer Center Institutional Review Board (IRB) and all patients provided written informed consent for tumor sequencing and review of patient medical records for detailed demographic, pathologic, and treatment information (NCT01775072). Detailed treatment history data were obtained for each patient and included all lines of systemic therapy from time of diagnosis of invasive carcinoma to the study data lock in February 2021. The exact regimen as well as the dates of start and stop of therapy were recorded for each treatment line.<br>To assess the effect of MAPK alterations on response to anti-HER2 therapy, we identified a subgroup of patients who received first line standard of care taxanes plus trastuzumab and pertuzumab (THP) and underwent tumor sequencing on a sample that was collected prior to start of therapy. The final cohort suitable for survival analysis included 145 patients.
Recruitment	The patients were recruited as per the prespecified inclusion and exclusion criteria. We are unaware of any major bias in recruitment other than the criteria outlined in the study protocol.
Ethics oversight	Memorial Sloan Kettering Cancer Center Institutional Review Board (IRB)

Note that full information on the approval of the study protocol must also be provided in the manuscript.

Clinical data

Policy information about clinical studies

All manuscripts should comply with the ICMJEguidelines for publication of clinical research and a completedCONSORT checklist must be included with all submissions.

Clinical trial registration	The study is registered under the clinicaltrials.gov identifier NCT01775072 https://clinicaltrials.gov/ct2/show/NCT01775072
Study protocol	NCT01775072
Data collection	A total of 733 breast tumor specimens from 664 patients with HER2+ metastatic breast cancer who underwent prospective clinical genomic profiling between April 2014 and February 2020
Outcomes	We did not pre-define outcomes.
